# 2,6-Bis[1-(2,6-diethyl­phenyl­imino)eth­yl]pyridine

**DOI:** 10.1107/S1600536808036842

**Published:** 2008-11-20

**Authors:** Yu-lin Yang, Rui-qing Fan, Wen-hui Li

**Affiliations:** a Department of Chemistry, Harbin Institute of Technology, Harbin 150001, People’s Republic of China; b College of Materials Science and Engineering, Harbin University of Science & Technology, Harbin 150040, People’s Republic of China

## Abstract

The title compound, C_29_H_35_N_3_, is the product of the condensation reaction between 2,6-diacetyl­pyridine and 2,6-diethyl­aniline. In the mol­ecule, the pyridyl ring is coplanar with the imino functional groups [torsion angles in the range 177.1 (2)–179.9 (2)°. The two 2,6-diethyl-substituted benzene rings are approximately perpendicular to the ethyl­idenepyridine central core, the dihedral angles being 88.7 (1) and 88.4 (1)°, respectively.

## Related literature

For applications of pyridine derivatives, see: Tang & VanSlyke (1987 ▶); Wang (2001 ▶). For the synthesis of the title mol­ecule, see: Fan *et al.* (2004 ▶). For structures of other imino derivatives, see: Mentes *et al.* (2001 ▶); Huang *et al.* (2006 ▶).
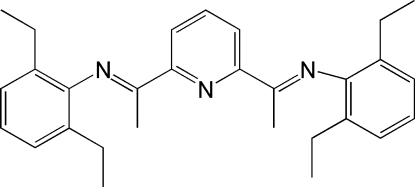


## Experimental

### 

#### Crystal data

C_29_H_35_N_3_
*M* *_r_* = 425.60
Monoclinic, 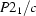

*a* = 7.9390 (8) Å
*b* = 12.3208 (13) Å
*c* = 25.998 (3) Å
β = 96.234 (2)°
*V* = 2528.0 (5) Å^3^
*Z* = 4
Mo *K*α radiation
μ = 0.07 mm^−1^
*T* = 193 (2) K
0.26 × 0.24 × 0.20 mm

#### Data collection

Bruker SMART APEX CCD area-detector diffractometer
Absorption correction: multi-scan (*SADABS*; Bruker, 1998 ▶) *T*_min_ = 0.983, *T*_max_ = 0.987
13906 measured reflections
4938 independent reflections
2362 reflections with *I* > 2σ(*I*)
*R*_int_ = 0.077

#### Refinement

*R*[*F* ^2^ > 2σ(*F* ^2^)] = 0.057
*wR*(*F* ^2^) = 0.101
*S* = 0.95
4938 reflections
289 parameters
H-atom parameters constrained
Δρ_max_ = 0.30 e Å^−3^
Δρ_min_ = −0.19 e Å^−3^

### 

Data collection: *SMART* (Bruker, 1998 ▶); cell refinement: *SAINT* (Bruker, 1998 ▶); data reduction: *SAINT*; program(s) used to solve structure: *SHELXS97* (Sheldrick, 2008 ▶); program(s) used to refine structure: *SHELXL97* (Sheldrick, 2008 ▶); molecular graphics: *SHELXTL* (Sheldrick, 2008 ▶); software used to prepare material for publication: *SHELXTL*.

## Supplementary Material

Crystal structure: contains datablocks global, I. DOI: 10.1107/S1600536808036842/bh2205sup1.cif

Structure factors: contains datablocks I. DOI: 10.1107/S1600536808036842/bh2205Isup2.hkl

Additional supplementary materials:  crystallographic information; 3D view; checkCIF report

## Figures and Tables

**Table 1:** Selected bond lengths (Å)

N1—C1	1.272 (3)
N1—C10	1.431 (3)
N2—C2	1.333 (2)
N2—C6	1.348 (2)
N3—C7	1.275 (2)
N3—C20	1.424 (3)

**Fig. 1.**
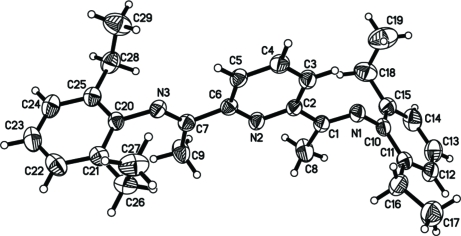
View of the molecule of (I) showing the atom-labelling scheme. Displacement ellipsoids are drawn at the 50% probability level.

**Fig. 2.**
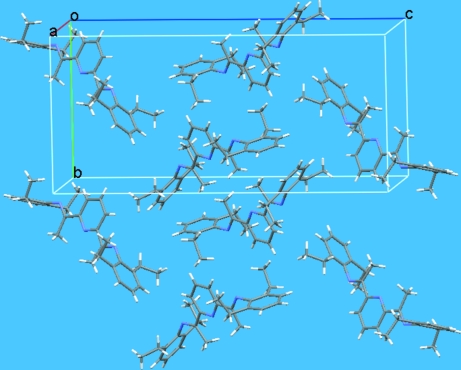
Packing of (I) along a cell axis direction.

## supplementary crystallographic information

### Comment

Luminescent coordination compounds based on pyridine-type ligands have attracted intensive attention due to their potential application in areas of sensor technologies and electro-luminescent devices (Tang & VanSlyke, 1987; Wang, 2001). In order to explore potential luminescent complexes of this type, we prepared a series of bis(iminoalkyl)pyridine ligands by the condensation of 2,6-diacetylpyridine with the corresponding aniline in methanol (Fan *et al.*, 2004). We report here the crystal structure of one of them, (I).

The molecular structure of (I) is shown in Fig. 1 and selected bond distances are given in Table 1. The pyridyl ring is coplanar with the two imino functional groups. The two imino C═N bonds have typical double-bond characteristics, with bond lengths of 1.272 (3) and 1.275 (2) Å, which are similar to that in BIP1, 1.266 (4) (Mentes *et al.*, 2001) and in 2,6-bis[1-(2,6-dimethylphenylimino)ethyl]pyridine, 1.265 (2) and 1.271 (2) Å (Huang *et al.*, 2006). Compound (I) possesses a structure which approximates *C*_s_ symmetry about a plane bisecting the central pyridyl ring. The two 2,6-diethyl-substituted phenyl rings are approximately perpendicular to the ethylidenepyridine ring, with the dihedral angles being 88.7° and 88.4°.

### Experimental

The title compound was synthesized according to the literature method of Fan *et al.* (2004). To a solution of 2,6-diethylpyridine (1.5 g, 9.2 mmol) in absolute methanol (40 ml) was added 2,6-diethylaniline (4.6 ml, 27.7 mmol). After the addition of several drops of formic acid, the reaction mixture was refluxed for 24 h and then allowed to cool down to room temperature. The crude product precipitated as a yellow powder. Pure (I) was obtained as yellow block crystals in 84% yield (3.3 g) upon recrystallization from methanol, giving single crystals suitable for X-ray diffraction.

### Refinement

The C-bound H atoms were positioned geometrically with C—H = 0.93–0.97 Å, and allowed to ride on their parent atoms with *U*_iso_(H) = 1.5*U*_eq_(carrier C) for methyl groups and *U*_iso_(H) = 1.2*U*_eq_(carrier C) otherwise.

### Crystal data

**Table:**

C_29_H_35_N_3_	*F*_000_ = 920
*M**_r_* = 425.60	*D*_x_ = 1.118 Mg m^−^^3^
Monoclinic, *P*2_1_/*c*	Mo *K*α radiation λ = 0.71073 Å
Hall symbol: -P 2ybc	Cell parameters from 13906 reflections
*a* = 7.9390 (8) Å	θ = 1.6–26.0º
*b* = 12.3208 (13) Å	µ = 0.07 mm^−^^1^
*c* = 25.998 (3) Å	*T* = 193 (2) K
β = 96.234 (2)º	Block, yellow
*V* = 2528.0 (5) Å^3^	0.26 × 0.24 × 0.20 mm
*Z* = 4	

### Data collection

**Table:**

Bruker SMART APEX CCD area-detector diffractometer	4938 independent reflections
Radiation source: fine-focus sealed tube	2362 reflections with *I* > 2σ(*I*)
Monochromator: graphite	*R*_int_ = 0.077
*T* = 193(2) K	θ_max_ = 26.0º
φ and ω scans	θ_min_ = 1.6º
Absorption correction: multi-scan(SADABS; Bruker, 1998)	*h* = −9→9
*T*_min_ = 0.983, *T*_max_ = 0.987	*k* = −15→12
13906 measured reflections	*l* = −31→32

### Refinement

**Table:**

Refinement on *F*^2^	Secondary atom site location: difference Fourier map
Least-squares matrix: full	Hydrogen site location: inferred from neighbouring sites
*R*[*F*^2^ > 2σ(*F*^2^)] = 0.057	H-atom parameters constrained
*wR*(*F*^2^) = 0.101	*w* = 1/[σ^2^(*F*_o_^2^) + (0.02*P*)^2^] where *P* = (*F*_o_^2^ + 2*F*_c_^2^)/3
*S* = 0.95	(Δ/σ)_max_ < 0.001
4938 reflections	Δρ_max_ = 0.30 e Å^−^^3^
289 parameters	Δρ_min_ = −0.19 e Å^−^^3^
Primary atom site location: structure-invariant direct methods	Extinction correction: none

### Fractional atomic coordinates and isotropic or equivalent isotropic displacement parameters (Å^2^)

**Table:**

	*x*	*y*	*z*	*U*_iso_*/*U*_eq_	
N1	−0.5544 (2)	0.61937 (15)	−0.15400 (7)	0.0377 (5)	
N2	−0.2647 (2)	0.69242 (15)	−0.04651 (7)	0.0342 (5)	
N3	0.0206 (2)	0.83861 (14)	0.04282 (7)	0.0328 (5)	
C1	−0.4636 (3)	0.60967 (19)	−0.11083 (9)	0.0353 (6)	
C2	−0.3645 (3)	0.70650 (19)	−0.09065 (9)	0.0315 (6)	
C3	−0.3771 (3)	0.80441 (18)	−0.11695 (9)	0.0379 (7)	
H3B	−0.4480	0.8113	−0.1477	0.045*	
C4	−0.2830 (3)	0.89137 (19)	−0.09692 (9)	0.0401 (7)	
H4A	−0.2893	0.9580	−0.1139	0.048*	
C5	−0.1790 (3)	0.87807 (18)	−0.05110 (9)	0.0346 (6)	
H5A	−0.1140	0.9355	−0.0367	0.041*	
C6	−0.1734 (3)	0.77816 (18)	−0.02709 (9)	0.0321 (6)	
C7	−0.0646 (3)	0.75836 (19)	0.02294 (9)	0.0328 (6)	
C8	−0.4473 (3)	0.50849 (18)	−0.07891 (9)	0.0551 (8)	
H8A	−0.5166	0.4524	−0.0959	0.083*	
H8B	−0.4837	0.5227	−0.0455	0.083*	
H8C	−0.3311	0.4854	−0.0748	0.083*	
C9	−0.0684 (3)	0.64705 (18)	0.04624 (9)	0.0516 (8)	
H9A	0.0057	0.6450	0.0780	0.077*	
H9B	−0.0314	0.5947	0.0225	0.077*	
H9C	−0.1818	0.6302	0.0531	0.077*	
C10	−0.6522 (3)	0.52973 (18)	−0.17576 (9)	0.0350 (6)	
C11	−0.5797 (3)	0.46024 (19)	−0.20928 (9)	0.0354 (6)	
C12	−0.6793 (3)	0.3774 (2)	−0.23284 (9)	0.0460 (7)	
H12A	−0.6339	0.3312	−0.2560	0.055*	
C13	−0.8439 (4)	0.3630 (2)	−0.22238 (10)	0.0529 (8)	
H13A	−0.9089	0.3071	−0.2382	0.063*	
C14	−0.9118 (3)	0.4313 (2)	−0.18850 (10)	0.0531 (8)	
H14A	−1.0227	0.4202	−0.1813	0.064*	
C15	−0.8197 (3)	0.5162 (2)	−0.16479 (10)	0.0442 (7)	
C16	−0.3966 (3)	0.4759 (2)	−0.21860 (9)	0.0542 (8)	
H16A	−0.3738	0.5533	−0.2186	0.065*	
H16B	−0.3261	0.4447	−0.1895	0.065*	
C17	−0.3415 (3)	0.4290 (2)	−0.26723 (10)	0.0677 (9)	
H17A	−0.2233	0.4437	−0.2685	0.102*	
H17B	−0.4061	0.4613	−0.2967	0.102*	
H17C	−0.3599	0.3520	−0.2677	0.102*	
C18	−0.8989 (3)	0.5920 (2)	−0.12862 (10)	0.0595 (8)	
H18A	−0.9946	0.5565	−0.1155	0.071*	
H18B	−0.8167	0.6094	−0.0994	0.071*	
C19	−0.9567 (4)	0.6936 (2)	−0.15592 (11)	0.0843 (11)	
H19A	−1.0065	0.7408	−0.1324	0.126*	
H19B	−1.0392	0.6764	−0.1845	0.126*	
H19C	−0.8616	0.7292	−0.1685	0.126*	
C20	0.1215 (3)	0.82874 (17)	0.09135 (9)	0.0314 (6)	
C21	0.2917 (3)	0.79922 (18)	0.09253 (10)	0.0366 (6)	
C22	0.3912 (3)	0.80093 (19)	0.14006 (11)	0.0481 (7)	
H22A	0.5045	0.7806	0.1417	0.058*	
C23	0.3256 (4)	0.8321 (2)	0.18473 (11)	0.0512 (8)	
H23A	0.3947	0.8347	0.2160	0.061*	
C24	0.1569 (4)	0.85952 (19)	0.18268 (10)	0.0466 (7)	
H24A	0.1129	0.8799	0.2130	0.056*	
C25	0.0504 (3)	0.85759 (18)	0.13639 (9)	0.0367 (6)	
C26	0.3694 (3)	0.76989 (19)	0.04370 (9)	0.0458 (7)	
H26A	0.2924	0.7225	0.0227	0.055*	
H26B	0.4738	0.7302	0.0530	0.055*	
C27	0.4070 (3)	0.86848 (19)	0.01206 (10)	0.0595 (8)	
H27A	0.4547	0.8456	−0.0185	0.089*	
H27B	0.4862	0.9146	0.0323	0.089*	
H27C	0.3040	0.9077	0.0024	0.089*	
C28	−0.1336 (3)	0.8865 (2)	0.13489 (9)	0.0482 (7)	
H28A	−0.1670	0.8812	0.1696	0.058*	
H28B	−0.1999	0.8342	0.1134	0.058*	
C29	−0.1747 (4)	0.9995 (2)	0.11419 (10)	0.0685 (9)	
H29A	−0.2938	1.0131	0.1142	0.103*	
H29B	−0.1447	1.0051	0.0795	0.103*	
H29C	−0.1117	1.0520	0.1357	0.103*	

### Atomic displacement parameters (Å^2^)

**Table:**

	*U*^11^	*U*^22^	*U*^33^	*U*^12^	*U*^13^	*U*^23^
N1	0.0406 (14)	0.0347 (13)	0.0358 (13)	−0.0017 (10)	−0.0047 (11)	−0.0046 (10)
N2	0.0406 (14)	0.0297 (12)	0.0312 (12)	−0.0026 (10)	−0.0010 (10)	−0.0022 (10)
N3	0.0371 (13)	0.0301 (12)	0.0309 (12)	−0.0024 (10)	0.0030 (10)	−0.0034 (10)
C1	0.0411 (17)	0.0318 (15)	0.0320 (15)	0.0018 (12)	−0.0001 (13)	−0.0012 (12)
C2	0.0369 (16)	0.0278 (15)	0.0293 (15)	0.0002 (12)	0.0020 (12)	−0.0022 (12)
C3	0.0455 (18)	0.0329 (15)	0.0340 (15)	−0.0013 (13)	−0.0009 (13)	−0.0007 (13)
C4	0.0536 (18)	0.0265 (15)	0.0396 (16)	−0.0012 (13)	0.0026 (14)	0.0026 (12)
C5	0.0408 (16)	0.0291 (15)	0.0337 (15)	−0.0042 (12)	0.0029 (13)	−0.0028 (12)
C6	0.0357 (16)	0.0269 (14)	0.0337 (15)	−0.0014 (12)	0.0044 (12)	−0.0011 (12)
C7	0.0369 (16)	0.0295 (15)	0.0318 (15)	−0.0001 (12)	0.0039 (13)	0.0007 (12)
C8	0.072 (2)	0.0370 (16)	0.0509 (18)	−0.0122 (15)	−0.0163 (16)	0.0078 (14)
C9	0.064 (2)	0.0381 (16)	0.0479 (17)	−0.0114 (14)	−0.0170 (15)	0.0096 (14)
C10	0.0386 (17)	0.0332 (15)	0.0311 (15)	−0.0026 (13)	−0.0057 (13)	0.0008 (12)
C11	0.0316 (16)	0.0389 (16)	0.0344 (15)	−0.0021 (13)	−0.0021 (13)	−0.0005 (13)
C12	0.053 (2)	0.0466 (17)	0.0372 (16)	−0.0019 (15)	−0.0017 (14)	−0.0079 (13)
C13	0.052 (2)	0.055 (2)	0.0492 (18)	−0.0161 (16)	−0.0035 (16)	−0.0064 (15)
C14	0.0335 (18)	0.071 (2)	0.0537 (19)	−0.0119 (16)	0.0004 (15)	−0.0030 (17)
C15	0.0375 (18)	0.0504 (18)	0.0439 (17)	0.0016 (14)	0.0000 (14)	−0.0038 (14)
C16	0.0444 (19)	0.071 (2)	0.0471 (18)	0.0011 (15)	0.0047 (14)	−0.0214 (15)
C17	0.063 (2)	0.087 (2)	0.054 (2)	0.0008 (18)	0.0091 (16)	−0.0043 (17)
C18	0.049 (2)	0.061 (2)	0.070 (2)	0.0079 (16)	0.0126 (16)	0.0072 (17)
C19	0.110 (3)	0.054 (2)	0.096 (3)	0.007 (2)	0.042 (2)	0.006 (2)
C20	0.0345 (16)	0.0256 (14)	0.0327 (15)	−0.0062 (12)	−0.0028 (13)	0.0009 (11)
C21	0.0391 (17)	0.0291 (15)	0.0410 (16)	−0.0041 (12)	0.0016 (14)	0.0062 (12)
C22	0.0389 (18)	0.0431 (17)	0.060 (2)	−0.0043 (13)	−0.0057 (16)	0.0111 (15)
C23	0.056 (2)	0.0497 (18)	0.0442 (19)	−0.0110 (16)	−0.0113 (16)	0.0052 (15)
C24	0.062 (2)	0.0439 (17)	0.0340 (16)	−0.0109 (15)	0.0035 (15)	−0.0012 (13)
C25	0.0407 (17)	0.0341 (15)	0.0351 (16)	−0.0057 (13)	0.0038 (14)	0.0008 (12)
C26	0.0416 (18)	0.0397 (16)	0.0567 (18)	0.0045 (13)	0.0072 (14)	0.0047 (14)
C27	0.070 (2)	0.0473 (18)	0.066 (2)	0.0092 (15)	0.0282 (17)	0.0120 (15)
C28	0.051 (2)	0.0554 (19)	0.0396 (16)	−0.0051 (15)	0.0118 (14)	−0.0067 (14)
C29	0.064 (2)	0.076 (2)	0.068 (2)	0.0192 (17)	0.0232 (17)	0.0168 (18)

### Geometric parameters (Å, °)

**Table:**

N1—C1	1.272 (3)	C16—H16A	0.9700
N1—C10	1.431 (3)	C16—H16B	0.9700
N2—C2	1.333 (2)	C17—H17A	0.9600
N2—C6	1.348 (2)	C17—H17B	0.9600
N3—C7	1.275 (2)	C17—H17C	0.9600
N3—C20	1.424 (3)	C18—C19	1.486 (3)
C1—C2	1.493 (3)	C18—H18A	0.9700
C1—C8	1.495 (3)	C18—H18B	0.9700
C2—C3	1.385 (3)	C19—H19A	0.9600
C3—C4	1.376 (3)	C19—H19B	0.9600
C3—H3B	0.9300	C19—H19C	0.9600
C4—C5	1.383 (3)	C20—C21	1.397 (3)
C4—H4A	0.9300	C20—C25	1.400 (3)
C5—C6	1.379 (3)	C21—C22	1.393 (3)
C5—H5A	0.9300	C21—C26	1.514 (3)
C6—C7	1.501 (3)	C22—C23	1.378 (3)
C7—C9	1.501 (3)	C22—H22A	0.9300
C8—H8A	0.9600	C23—C24	1.376 (3)
C8—H8B	0.9600	C23—H23A	0.9300
C8—H8C	0.9600	C24—C25	1.394 (3)
C9—H9A	0.9600	C24—H24A	0.9300
C9—H9B	0.9600	C25—C28	1.500 (3)
C9—H9C	0.9600	C26—C27	1.515 (3)
C10—C11	1.390 (3)	C26—H26A	0.9700
C10—C15	1.399 (3)	C26—H26B	0.9700
C11—C12	1.391 (3)	C27—H27A	0.9600
C11—C16	1.511 (3)	C27—H27B	0.9600
C12—C13	1.375 (3)	C27—H27C	0.9600
C12—H12A	0.9300	C28—C29	1.515 (3)
C13—C14	1.370 (3)	C28—H28A	0.9700
C13—H13A	0.9300	C28—H28B	0.9700
C14—C15	1.383 (3)	C29—H29A	0.9600
C14—H14A	0.9300	C29—H29B	0.9600
C15—C18	1.511 (3)	C29—H29C	0.9600
C16—C17	1.498 (3)		
			
C1—N1—C10	120.5 (2)	C16—C17—H17A	109.5
C2—N2—C6	117.7 (2)	C16—C17—H17B	109.5
C7—N3—C20	121.0 (2)	H17A—C17—H17B	109.5
N1—C1—C2	117.5 (2)	C16—C17—H17C	109.5
N1—C1—C8	125.1 (2)	H17A—C17—H17C	109.5
C2—C1—C8	117.4 (2)	H17B—C17—H17C	109.5
N2—C2—C3	122.9 (2)	C19—C18—C15	110.6 (2)
N2—C2—C1	116.1 (2)	C19—C18—H18A	109.5
C3—C2—C1	121.0 (2)	C15—C18—H18A	109.5
C4—C3—C2	119.0 (2)	C19—C18—H18B	109.5
C4—C3—H3B	120.5	C15—C18—H18B	109.5
C2—C3—H3B	120.5	H18A—C18—H18B	108.1
C3—C4—C5	118.8 (2)	C18—C19—H19A	109.5
C3—C4—H4A	120.6	C18—C19—H19B	109.5
C5—C4—H4A	120.6	H19A—C19—H19B	109.5
C6—C5—C4	118.9 (2)	C18—C19—H19C	109.5
C6—C5—H5A	120.6	H19A—C19—H19C	109.5
C4—C5—H5A	120.6	H19B—C19—H19C	109.5
N2—C6—C5	122.7 (2)	C21—C20—C25	121.7 (2)
N2—C6—C7	115.6 (2)	C21—C20—N3	119.4 (2)
C5—C6—C7	121.7 (2)	C25—C20—N3	118.7 (2)
N3—C7—C6	117.1 (2)	C22—C21—C20	118.0 (2)
N3—C7—C9	125.3 (2)	C22—C21—C26	120.3 (2)
C6—C7—C9	117.6 (2)	C20—C21—C26	121.7 (2)
C1—C8—H8A	109.5	C23—C22—C21	121.5 (3)
C1—C8—H8B	109.5	C23—C22—H22A	119.3
H8A—C8—H8B	109.5	C21—C22—H22A	119.3
C1—C8—H8C	109.5	C24—C23—C22	119.4 (3)
H8A—C8—H8C	109.5	C24—C23—H23A	120.3
H8B—C8—H8C	109.5	C22—C23—H23A	120.3
C7—C9—H9A	109.5	C23—C24—C25	121.7 (3)
C7—C9—H9B	109.5	C23—C24—H24A	119.1
H9A—C9—H9B	109.5	C25—C24—H24A	119.1
C7—C9—H9C	109.5	C24—C25—C20	117.7 (2)
H9A—C9—H9C	109.5	C24—C25—C28	121.1 (2)
H9B—C9—H9C	109.5	C20—C25—C28	121.2 (2)
C11—C10—C15	121.4 (2)	C21—C26—C27	112.7 (2)
C11—C10—N1	118.6 (2)	C21—C26—H26A	109.0
C15—C10—N1	119.9 (2)	C27—C26—H26A	109.0
C10—C11—C12	118.2 (2)	C21—C26—H26B	109.0
C10—C11—C16	119.5 (2)	C27—C26—H26B	109.0
C12—C11—C16	122.2 (2)	H26A—C26—H26B	107.8
C13—C12—C11	121.0 (3)	C26—C27—H27A	109.5
C13—C12—H12A	119.5	C26—C27—H27B	109.5
C11—C12—H12A	119.5	H27A—C27—H27B	109.5
C14—C13—C12	119.8 (3)	C26—C27—H27C	109.5
C14—C13—H13A	120.1	H27A—C27—H27C	109.5
C12—C13—H13A	120.1	H27B—C27—H27C	109.5
C13—C14—C15	121.7 (3)	C25—C28—C29	113.5 (2)
C13—C14—H14A	119.2	C25—C28—H28A	108.9
C15—C14—H14A	119.2	C29—C28—H28A	108.9
C14—C15—C10	117.9 (2)	C25—C28—H28B	108.9
C14—C15—C18	120.7 (3)	C29—C28—H28B	108.9
C10—C15—C18	121.4 (2)	H28A—C28—H28B	107.7
C17—C16—C11	117.4 (2)	C28—C29—H29A	109.5
C17—C16—H16A	108.0	C28—C29—H29B	109.5
C11—C16—H16A	108.0	H29A—C29—H29B	109.5
C17—C16—H16B	108.0	C28—C29—H29C	109.5
C11—C16—H16B	108.0	H29A—C29—H29C	109.5
H16A—C16—H16B	107.2	H29B—C29—H29C	109.5
			
C10—N1—C1—C2	179.8 (2)	C12—C13—C14—C15	1.0 (4)
C10—N1—C1—C8	0.3 (4)	C13—C14—C15—C10	−1.0 (4)
C6—N2—C2—C3	0.2 (3)	C13—C14—C15—C18	178.4 (2)
C6—N2—C2—C1	−179.9 (2)	C11—C10—C15—C14	−0.2 (4)
N1—C1—C2—N2	−177.1 (2)	N1—C10—C15—C14	177.2 (2)
C8—C1—C2—N2	2.4 (3)	C11—C10—C15—C18	−179.6 (2)
N1—C1—C2—C3	2.8 (3)	N1—C10—C15—C18	−2.3 (4)
C8—C1—C2—C3	−177.7 (2)	C10—C11—C16—C17	−158.5 (2)
N2—C2—C3—C4	0.0 (4)	C12—C11—C16—C17	22.1 (4)
C1—C2—C3—C4	−179.9 (2)	C14—C15—C18—C19	−98.9 (3)
C2—C3—C4—C5	0.0 (4)	C10—C15—C18—C19	80.6 (3)
C3—C4—C5—C6	−0.1 (3)	C7—N3—C20—C21	91.8 (3)
C2—N2—C6—C5	−0.3 (3)	C7—N3—C20—C25	−93.3 (3)
C2—N2—C6—C7	179.4 (2)	C25—C20—C21—C22	−1.1 (3)
C4—C5—C6—N2	0.3 (3)	N3—C20—C21—C22	173.6 (2)
C4—C5—C6—C7	−179.4 (2)	C25—C20—C21—C26	−178.9 (2)
C20—N3—C7—C6	177.2 (2)	N3—C20—C21—C26	−4.2 (3)
C20—N3—C7—C9	−1.0 (4)	C20—C21—C22—C23	−0.9 (4)
N2—C6—C7—N3	−178.6 (2)	C26—C21—C22—C23	177.0 (2)
C5—C6—C7—N3	1.1 (3)	C21—C22—C23—C24	1.7 (4)
N2—C6—C7—C9	−0.2 (3)	C22—C23—C24—C25	−0.6 (4)
C5—C6—C7—C9	179.5 (2)	C23—C24—C25—C20	−1.3 (4)
C1—N1—C10—C11	−91.4 (3)	C23—C24—C25—C28	179.4 (2)
C1—N1—C10—C15	91.2 (3)	C21—C20—C25—C24	2.1 (3)
C15—C10—C11—C12	1.4 (3)	N3—C20—C25—C24	−172.6 (2)
N1—C10—C11—C12	−175.95 (19)	C21—C20—C25—C28	−178.5 (2)
C15—C10—C11—C16	−178.0 (2)	N3—C20—C25—C28	6.8 (3)
N1—C10—C11—C16	4.6 (3)	C22—C21—C26—C27	−100.8 (3)
C10—C11—C12—C13	−1.5 (4)	C20—C21—C26—C27	77.0 (3)
C16—C11—C12—C13	177.9 (2)	C24—C25—C28—C29	102.6 (3)
C11—C12—C13—C14	0.3 (4)	C20—C25—C28—C29	−76.7 (3)
